# DH82 Canine and RAW264.7 Murine Macrophage Cell Lines Display Distinct Activation Profiles Upon Interaction With *Leishmania infantum and Leishmania amazonensis*

**DOI:** 10.3389/fcimb.2020.00247

**Published:** 2020-06-12

**Authors:** Natalia Rocha Nadaes, Leandro Silva da Costa, Raissa Couto Santana, Isabel Ferreira LaRocque-de-Freitas, Áislan de Carvalho Vivarini, Deivid Costa Soares, Amanda Brito Wardini, Ulisses Gazos Lopes, Elvira M. Saraiva, Celio Geraldo Freire-de-Lima, Debora Decote-Ricardo, Lucia Helena Pinto-da-Silva

**Affiliations:** ^1^Instituto de Veterinária, Universidade Federal Rural Do Rio de Janeiro, Seropédica, Brazil; ^2^Instituto de Bioquímica Médica Leopoldo De Meis, Universidade Federal Do Rio de Janeiro, Rio de Janeiro, Brazil; ^3^Instituto de Biofísica Carlos Chagas Filho, Universidade Federal Do Rio de Janeiro, Rio de Janeiro, Brazil; ^4^Instituto de Microbiologia Paulo de Góes, Universidade Federal Do Rio de Janeiro, Rio de Janeiro, Brazil

**Keywords:** DH82 canine, RAW 264.7 murine, macrophages, Leishmania infection, oxidative burst, cytokines, arginase activity

## Abstract

Leishmaniasis is an anthropozoonotic disease, and dogs are considered the main urban reservoir of the parasite. Macrophages, the target cells of *Leishmania sp*., play an important role during infection. Although dogs have a major importance in the epidemiology of the disease, the majority of the current knowledge about *Leishmania*–macrophage interaction comes from murine experimental models. To assess whether the canine macrophage strain DH82 is an accurate model for the study of *Leishmania* interaction, we compared its infection by two species of *Leishmania* (*Leishmania infantum* and *L. amazonensis*) with the murine macrophage cell line (RAW264.7). Our results demonstrated that *L. amazonensis* survival was around 40% at 24 h of infection inside both macrophage cell lines; however, a reduction of 4.3 times in *L. amazonensis* infection at 48 h post-infection in RAW 264.7 macrophages was observed. The survival index of *L. infantum* in DH82 canine macrophages was around 3 times higher than that in RAW264.7 murine cells at 24 and 48 h post-infection; however, at 48 h a reduction in both macrophages was observed. Surprisingly at 24 h post-infection, NO and ROS production by DH82 canine cells stimulated with LPS or menadione or during *Leishmania* infection was minor compared to murine RAW264.7. However, basal arginase activity was higher in DH82 cells when compared to murine RAW264.7 cells. Analysis of the cytokines showed that these macrophages present a different response profile. *L. infantum* induced IL-12, and *L. amazonensis* induced IL-10 in both cell lines. However, *L. infantum and L. amazonensis* also induced TGF-β in RAW 264.7. CD86 and MHC expression showed that *L. amazonensis* modulated them in both cell lines. Conversely, the parasite load profile did not show significant difference between both macrophage cell lines after 48 h of infection, which suggests that other mechanisms of *Leishmania* control could be involved in DH82 cells.

## Introduction

Leishmaniasis is an anthropozoonotic disease transmitted by sandflies, presenting a wide array of clinical symptoms, ranging from cutaneous to visceral manifestations. Dogs are considered the main reservoir of the disease, which is present in different countries of the Old and New World, in urban and periurban areas (Dantas-Torres et al., 2012). Canine leishmaniasis is a complex disease with variable clinical signs and different degrees of susceptibility caused mainly by *Leishmania infantum*. It is considered a serious public health problem since *L. infantum* is the causative agent of human visceral leishmaniasis, the more severe form of this disease. However, other species have also been reported in dogs, such as *L. amazonensis, L. mexicana*, and *L. braziliensis* (Gontijo and De Carvalho, 2003; Castro et al., 2007; Valdivia et al., 2017; Paz et al., 2018).

Macrophages are important innate immune cells in the defense against intracellular microorganisms and play an important role in *Leishmania* infection, since they are the target cells infected by the parasite. Superoxide anion (${\text{O}}_{2}^{-}$) and nitric oxide (NO) are critical molecules in controlling *Leishmania* infection and are produced by macrophages in response to *Leishmania* phagocytosis and after the activation of macrophages by interferon (IFN)-γ and tumor necrosis factor (TNF)-α (Channon et al., 1984; Gantt et al., 2001; Horta et al., 2012).

The majority of the current knowledge about *Leishmania*–macrophage interaction comes from murine experimental models using different macrophage sources, such as peritoneal, bone marrow-derived, or murine cell lineages (Mosser and Rosenthal, 1993; Handman and Bullen, 2002; Naderer and McConville, 2008). The use of a canine macrophage cell line to study the early aspects of *Leishmania* interaction is easier and more practical compared to macrophages obtained from the bone marrow or purified from peripheral blood from dogs. Added to that, the use of a cell line from an animal species that is the parasite reservoir can contribute for a better understanding about infection and cell response differences among murine and canine experimental models.

Some works have reported the use of DH82, a canine macrophage cell line, as a model to study infection by different microorganisms like virus, bacteria, and protozoa (Howerth et al., 2004; Maia et al., 2007; Ponnusamy and Clinkenbeard, 2012; Mendonça et al., 2017). In this study, we evaluated the interaction of DH82 canine macrophages by *L. amazonensis*, the causative agent of a cutaneous and diffuse cutaneous disease, and *L. infantum*, the causative agent of visceral disease, analyzing ROS and NO production, cytokine profile, arginase activity, and surface molecule expression in comparison to RAW264.7 murine macrophages, a cell line widely used in experimental studies. Our results show that DH82 cells have different characteristics in relation to the immune response to *Leishmania* compared to RAW264.7 ones.

## Materials and Methods

### Parasites

*Leishmania infantum* (MCAN/BR/2008/OP46) isolated from an infected dog from Governador Valadares, Minas Gerais State, Brazil, characterized by molecular techniques and hamster infection (Moreira et al., 2012, 2016), and *Leishmania amazonensis* (MHOM/BR/75/Josefa) were maintained at 26°C in Schneider's insect medium (Sigma) supplemented with 10% heat-inactivated fetal calf serum (Cripion), 10% human urine, and 40 μg mL^−1^ gentamicin (Sigma). In all assays, promastigotes in the stationary phase of growth (5–6 days) were washed twice in phosphate-buffered saline (PBS) pH 7.2) at 2,700 × *g* for 13 min at 22°C, resuspended in PBS, and used throughout.

### Macrophages

Murine RAW264.7 macrophages were maintained in RPMI 1640 medium (Sigma) supplemented with 10% FBS (Cripion) at 37°C 5% CO_2_. Canine DH82 macrophages, kindly supplied by Prof. Marcelo Bahia Labruna (Faculdade de Medicina Veterinária e Zootecnia, Universidade de São Paulo, SP, Brazil), were cultured in DMEM (Sigma) supplemented with 10% FBS (Cripion) at 37°C 5% CO_2_.

### Macrophage Interaction Assays

Macrophages (2 × 10^5^/well) adhered to coverslips (Vision Glass) in 24-well plates (Corning, USA) were incubated for 2 h at 37°C 5% CO_2_ and then infected with *Leishmania* promastigotes in the ratio of 5 parasites: 1 macrophage. Macrophages interacting with *L. amazonensis* were maintained at 34°C 5% CO_2_, whereas cells interacting with *L. infantum* were maintained at 37°C 5% CO_2_. After 2 h of infection, some cultures were washed twice with PBS, fixed, and stained using Diff-Quick (Laborclin) and the association index (uptake) evaluated. To determine the infection index, the cultures were kept in culture for 16 h. After rinsing with PBS twice, the cultures were maintained for an additional 24 and 48 h in the same conditions as above. Then, the cells were washed twice in PBS, fixed, and stained as described above. The association (2 h post-infection) and survival (24 or 48 h post-infection) indexes were assessed by multiplying the percentage of infected macrophages by the number of parasites per macrophage through randomly counting at least 200 cells in each of triplicate coverslips.

### Parasite Load

After 48 h of infection, macrophage cell monolayers were washed and incubated with 0.01% SDS (Sigma) for 10 min to allow lysis of macrophages and the release of amastigotes. Then, cultures were fed with 1 mL of Schneider's medium, supplemented with 10% FCS, and kept at 27°C for an additional 2 days. The relative intracellular load of *L. amazonensis* and *L. infantum* amastigotes was measured by assessing the number of extracellular motile promastigotes transformed (Gomes et al., 2000; Ribeiro-Gomes et al., 2006, 2007).

### Detection of Nitric Oxide and ROS Production in DH82 Canine and RAW264.7 Murine Macrophages

The amount of intracellular nitric oxide (NO), total reactive oxygen species (tROS), and mitochondrial ROS (mitROS) was assessed through fluorescent probes. Macrophages (10^6^) were plated in 6-well tissue culture plates, stimulated or not with LPS (1 μg/mL) or menadione (0.05 mM) (positive controls) or infected with *L. amazonensis* or *L. infantum* (5 parasites: 1 macrophage). The cultures were maintained for 24 h at 34°C or 37°C/5% CO_2_ according to the parasite species. Then, the cells were washed to remove free parasites and were labeled with 1 μM DAF-FM (Molecular Probes), 0.5 μM H_2_DCFDA (Thermo Fisher), or 0.25 μM MITOsox (Thermo Fisher). After 45 min in the dark at 37°C, the cells were washed and incubated for more 15 min under the same conditions. Cells were then mechanically released using cell scraper and resuspended in 400 μL of PBS. Stained cells (without any stimuli) were used as negative control to normalize the data in relation to stimulated or infected cells. The percentages of DAF-, DCF-, or MITOsox-positive cells were analyzed by flow cytometry (FACSCalibur) after acquiring 10,000 events. The analyses were performed on the CellQuest program. The gating strategy used in the cytometry evaluation was only to identify a homogeneous macrophages population, since we are working with a culture cell line (Figure S1).

### Quantitative Real-Time RT-PCR

Total RNA of RAW264.7 and DH82 cells (1 × 10^6^ cells) was extracted with RNeasy Plus Mini Kit (Qiagen 74134, Germany), and 1 μg aliquot was reverse transcribed to first-strand cDNA with ImProm-II (Promega) and oligo(dT) primer according to the manufacturer's instructions. The DNA sequences of the primers used were IL-12-F (mouse): 5′-TCAAGAGCAGTAGCAGTTCCCCTG-3′, IL-12-R (mouse): 5′-GGTCCAGTGTGACCTTCTCTGCA-3′, IL-12-F (dog): 5′-GCGTCTTCCCTCATGACC-3′, IL-12-R (dog): 5′-GGGTGCCAGTCCAACTCTAC-3′, IL-10-F (mouse): 5′-CCCAGAAATCAAGGAGCATT-3′, IL-10-R (mouse): 5′-TCACTCTTCACCTGCTCCAC-3′, IL-10-F (dog): 5′-GGTTGCCAGCCCTGTCGG-3′ IL-10-R (dog): 5′-GCGTCGCAGCCTCAGTCTCA-3′, IL-6-F (dog): 5′-GGGAAAGCAGTAGCCATCAC-3′, IL-6-R (dog): 5′-CAGGACCCCAGCTATGAACT-3′, TGF-β-F (mouse): 5′-GTGGCTGAACCAAGGAGACGG-3′, TGF-β-R (mouse): 5′-GGCTGATCCCGTTGATTTCCACG-3′, TGF-β-F (dog): 5′-CGAAGCCCTCGACTCC-3′, TGF-β-R (dog): 5′-TGGCTGYCCTTTGATGTCAC-3′, GAPDH-F: 5′-TGCACCACCAACTGCTTAGC-3′ and GAPDH-R: 5′-GGCATGGACTGTGGTCATGAG-3′. qRT-PCR data from the experiments were normalized using Gapdh primers as an endogenous control. Amplicon specificity was carefully verified by the presence of a single melting temperature peak in dissociation curves run after real-time RT-PCR. Real-time quantitative RT-PCR (qRT-PCR) was performed via the Applied Biosystems StepOne™ detection system (Applied Biosystems) using GoTaq® qPCR Master Mix (Promega Corp., Madison, WI, USA). All expression ratios were computed via the analysis of relative gene expression ΔΔCt method through the StepOne software version 2.0 (Applied Biosystems).

### Arginase Activity

Uninfected or infected macrophage cell lines stimulated or not with 40 ng/mL of murine interleukin-4 (IL-4, eBioscience) for 24 h were lysed to determine the arginase activity. The macrophage lysates were obtained from 2.5 × 10^5^ cells treated with 100 μL of 0.1% Triton X-100 for 30 min, followed by the addition of 100 μL of a buffer containing 25 mM Tris–HCl (pH 7.4) and 10 μL of 10 mM MnSO_4_. The enzyme was then activated by heating for 10 min at 56°C, and arginine hydrolysis was carried out by incubating 100 μL of the activated lysate with 100 μL of 0.1 M arginine (pH 9.7) at 37°C for 1 h. The reaction was stopped with 800 μL of H_2_SO_4_-H_3_PO_4_-H_2_O (1:3:7 [vol/vol/vol]) and 40 μL of 10% α-isonitrosopropiophenone in 100% methanol and heated to 100°C for 30 min. The urea concentration was measured at 540 nm. One unit of enzyme activity was defined as the amount that catalyzed the formation of 1 μmol of urea per min.

### MHC and CD86 Expression

The cells were infected or not with *L. infantum and L. amazonensis* and were activated or not by LPS (500 ng/mL). After 24 h post-infection, the cells were detached, washed, adjusted to a concentration of 5.0 × 10^5^ cells/tube, and incubated with blocking buffer (CD16/CD32 Fc Block—eBioscience) for 15 min on ice to prevent non-specific antibody binding to Fc receptors. Cells were stained with anti-dog MHC class II-FITC (Serotech), anti-mouse MHC class II-FITC, and anti-mouse CD86-PECy5 (eBioscience). All washing steps were performed with PBS containing 3% FCS and 0.02% sodium azide. Data were acquired (10,000 events), evaluated on FACSCalibur™ cytometer and analyzed using CellQuest® software (BD Biosciences, Heidelberg, Germany).

### Statistical Analysis

Data were analyzed using Student's *t*-test for comparison of two groups or ANOVA for more than three groups with Tukey post-test. Analyses were performed using the GraphPad Prism 5.0 software. The *p*-value was considered significant when equal to or <0.05.

## Results

**Figure 1:**
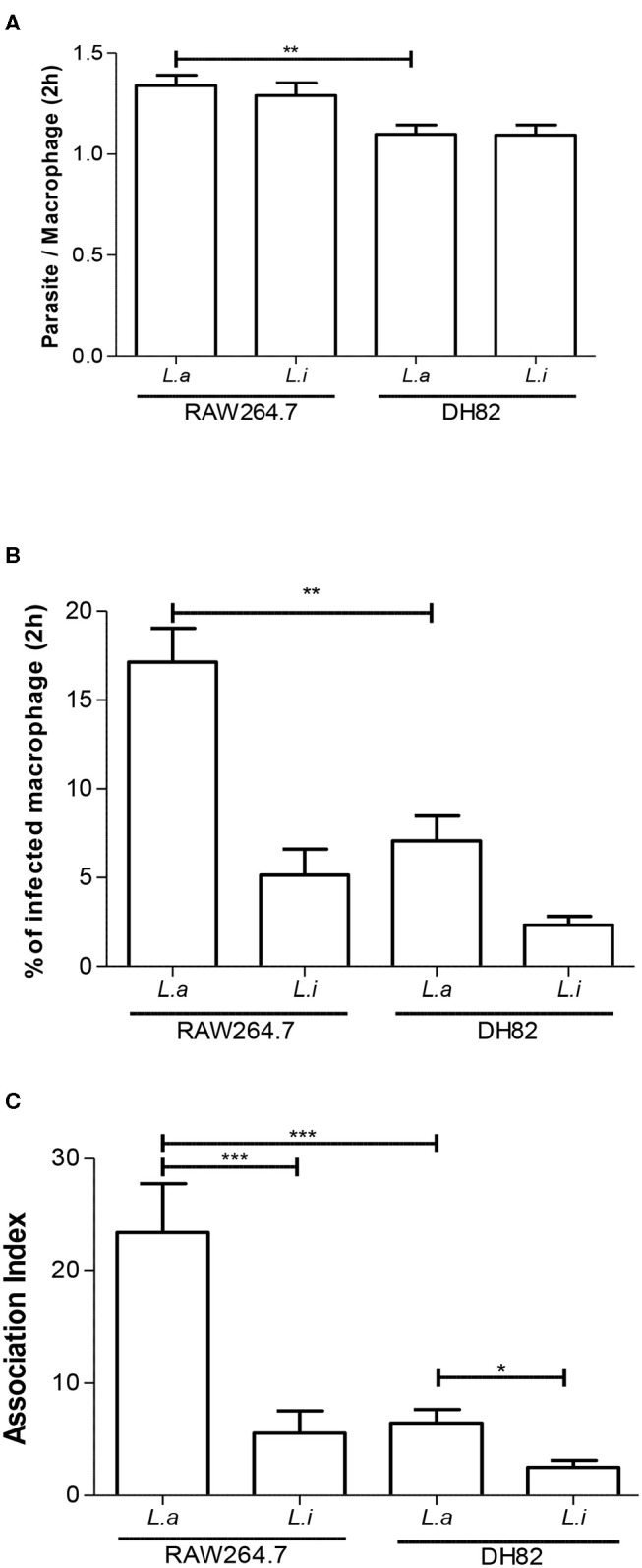
Association index of *Leishmania* in macrophages. Parasite per macrophages **(A)**, % infected macrophages **(B)**, and association index **(C)** in canine DH82 and murine RAW264.7. Macrophages were incubated with *L. amazonensis* (L.a) and *L. infantum* (L.i) promastigotes (5 parasites: 1 macrophage) for 2 h_._ The data represent mean ± SEM of three independent experiments done in triplicates. ^***^p < 0.0001; ^**^p = 0.0093; ^*^p = 0.005.

**Figure 2:**
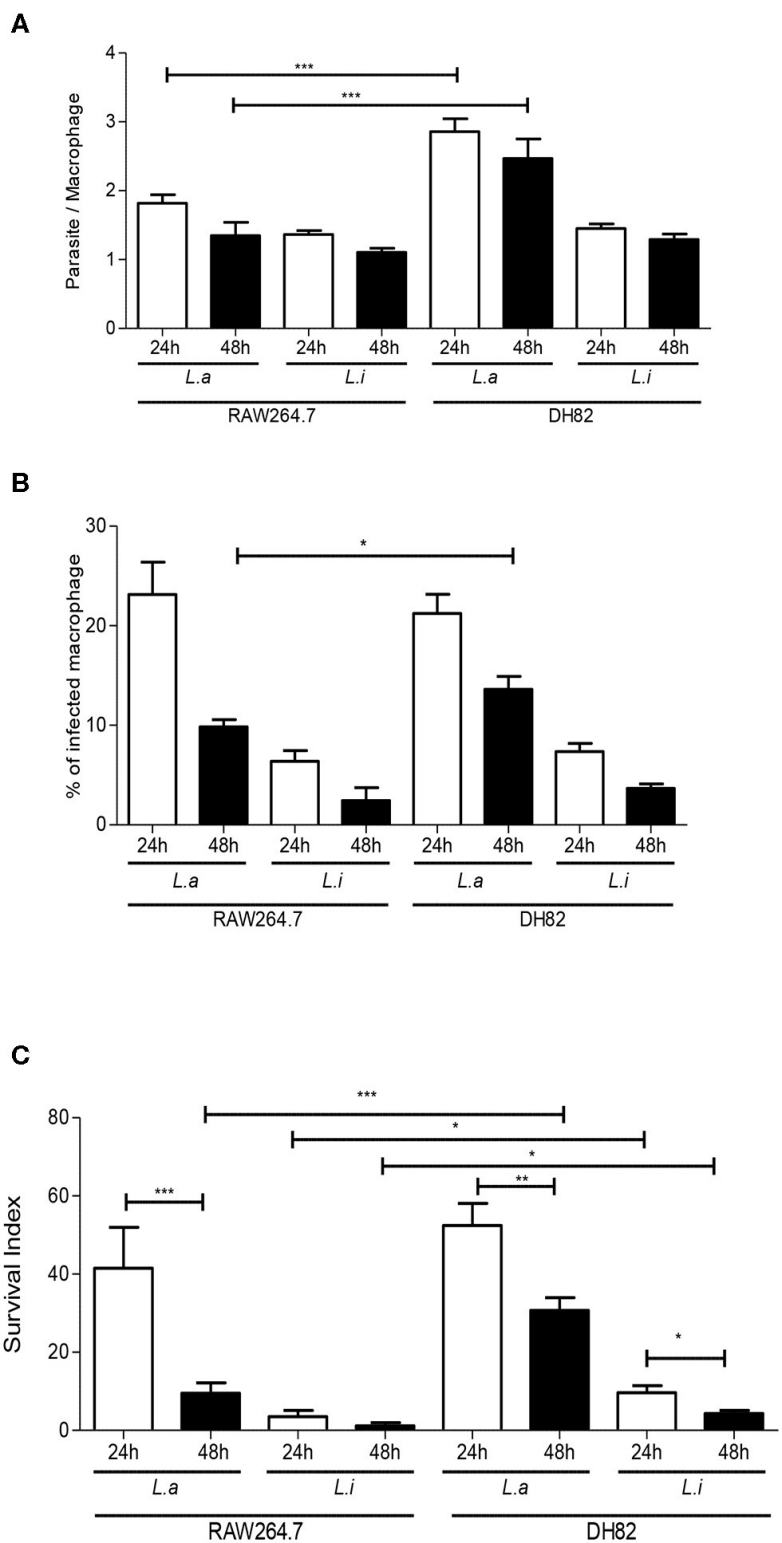
*Leishmania* interaction with macrophages. Parasite per macrophages **(A)**, % infected-macrophages **(B)**, and survival index **(C)** in canine DH82 and murine RAW264.7. Macrophages were infected with *L. amazonensis* (L.a) and *L. infantum* (L.i) promastigotes (5 parasites: 1 macrophage) for 24 and 48 h. The data represent mean ± SEM of six independent experiments done in triplicates. ^**^p < 0.008; ^*^p < 0.05; ^***^p < 0.0001.

First, we incubated DH82 canine and RAW264.7 murine macrophages with *L. amazonensis* or *L. infantum* promastigotes for 2 h, and the capacity of both species to interact with these host cells was evaluated. Our results showed that the number of parasites per cell and the % of infected macrophages was 1.2 and 2.4 times higher in *L. amazonensis* infected RAW264.7 compared to DH82 (Figures 1A,B). The association index showed that *L. amazonensis* interacts 2.4 times and 4.2 times more than *L. infantum* promastigotes with DH82 and RAW264.7 macrophages, respectively (Figure 1C). Assessing the parasite number per cell of *L. amazonensis* in DH82, there is no significant difference in the number of parasite per macrophages at 24 and 48 h of interaction, although we observed that the parasite number per cell was 1.5 and 1.8 times higher than in RAW264.7 in 24 and 48 h (Figure 2A, Figure S2). On the other hand, the % of infected DH82 macrophages by *L. amazonensis* was 1.3 times higher at the 48 h than in RAW264.7 (Figure 2B). In *L. infantum* survival, no significant difference was observed among DH82 or RAW264.7 macrophages, neither in the percentage of infected macrophages nor in the number of parasites per cell in 24 or 48 h post-infection infection (Figures 2A,B). Evaluating *Leishmania* survival in DH82 canine macrophages, we demonstrated that *L. amazonensis* infection decreased 1.6 times 48 h post-infection (Figure 2C). Comparing to *L. infantum, L. amazonensis* survival was 5 and 7.2 times higher, respectively, at 24 and 48 h post-infection (Figure 2C). A decrease of 1.6 and 2.3 times in the parasite survival after 48 h post-infection was observed for *L. amazonensis* and *L. infantum*, respectively.

**Figure 3:**
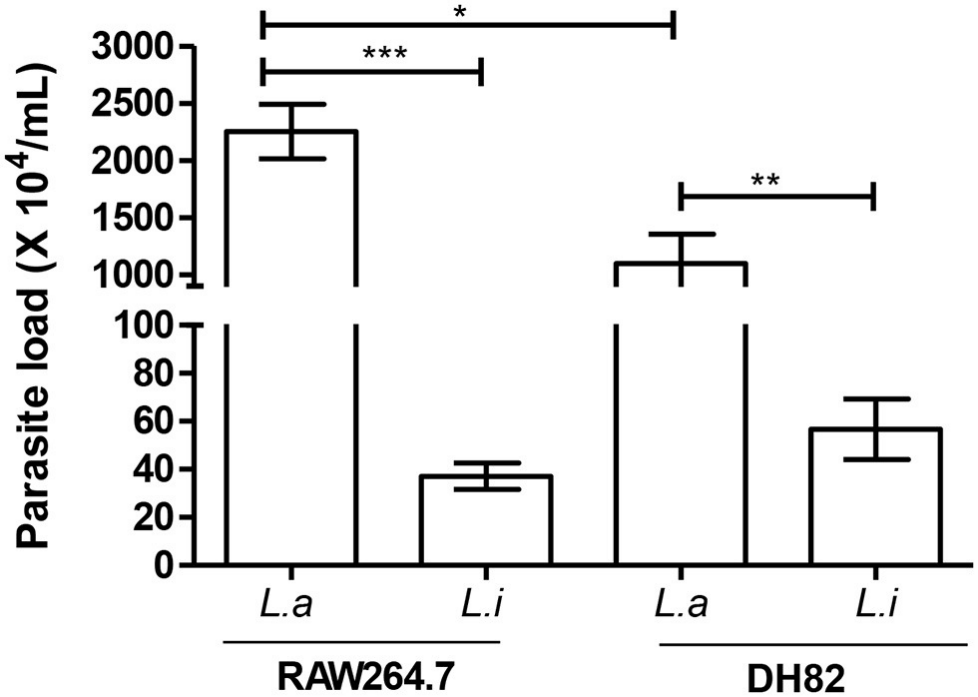
Parasite load in infected macrophages. Canine (DH82) and murine (RAW264.7) macrophages were infected for 48 h at 34°C or 37°C/5% CO_2_, according to the *Leishmania* species. Then, the cells were washed, lysed, and fed with Schneider's medium. Macrophage parasite load was evaluated after 2 days in culture at 27°C by extracellular promastigotes production. Results from three independent experiments in triplicate ± SEM. *L. amazonensis* (L.a) and *L. infantum* (L.i). *p < 0.05; **p = 0.0043; ***p < 0.0009.

Comparing parasite survival in RAW264.7 murine macrophages at 24 h post-infection, 42% of *L. amazonensis* survival was observed, similarly to the same time point in the DH82 canine macrophages; however, it decreased 4.3 times at 48 h in RAW264.7 (Figure 2C). In RAW264.7 macrophages, *L. infantum* survival did not show significant difference 24 and 48 h post-infection (Figure 2C). The interaction of *L. amazonensis* in DH82 canine macrophages was 1.2 and 3.2 times higher than in RAW264.7 murine macrophages at 24 and 48 h post-infection, respectively. A similar difference was observed in the interaction of *L. infantum* that was 3 and 3.5 times lower in RAW264.7 murine macrophages than in DH82 canine macrophages at 24 and 48 h, respectively. *L. infantum* parasite load after 48 h of infection was not statistically different in both macrophages, and *L. amazonensis* parasite load was twice higher in RAW264.7 than in DH82 cells (Figure 3).

**Figure 4:**
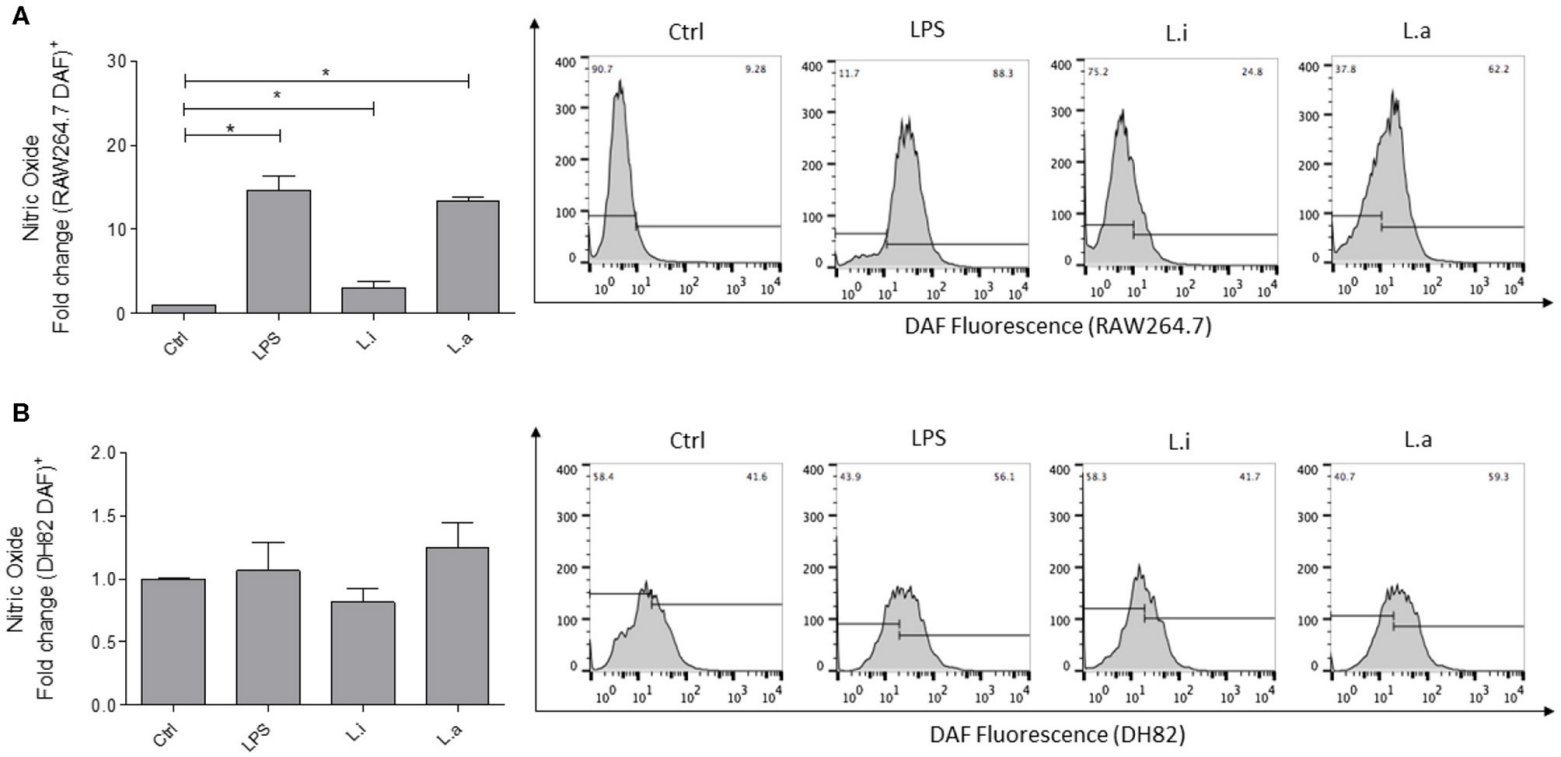
Intracellular nitric oxide (NO) production in murine (RAW264.7) and canine (DH82) macrophages. RAW264.7 **(A)** and DH82 **(B)** macrophages were stimulated with 1 μg/mL LPS or infected with 5p:1 m of *L. infantum* (L.i) or *L. amazonensis* (L.a) and NO production measured using DAF-FM. Data were normalized as fold change in comparison to control cells (unstimulated cells incubate with DAF) and are presented as mean ± SEM of three independent experiments. *p < 0.05. Representative histograms of %DAF-FM^+^ cells in unstimulated cells (Ctrl), stimulated with LPS, or infected with *L. infantum* or *L. amazonensis* are shown.

Then, we compared the ability of both cell lines to produce ROS and NO during *Leishmania* infection. Our results showed that DH82 canine macrophages did not augment the NO expression, when the cells were stimulated with LPS or infected with *L. infantum* or *L. amazonensis* (Figure 4B). However, an increase of 14.6, 3.0, and 13.4 times in NO production by RAW264.7 stimulated with LPS, *L. infantum*, and *L. amazonensis*, respectively, was observed (Figure 4A).

**Figure 5:**
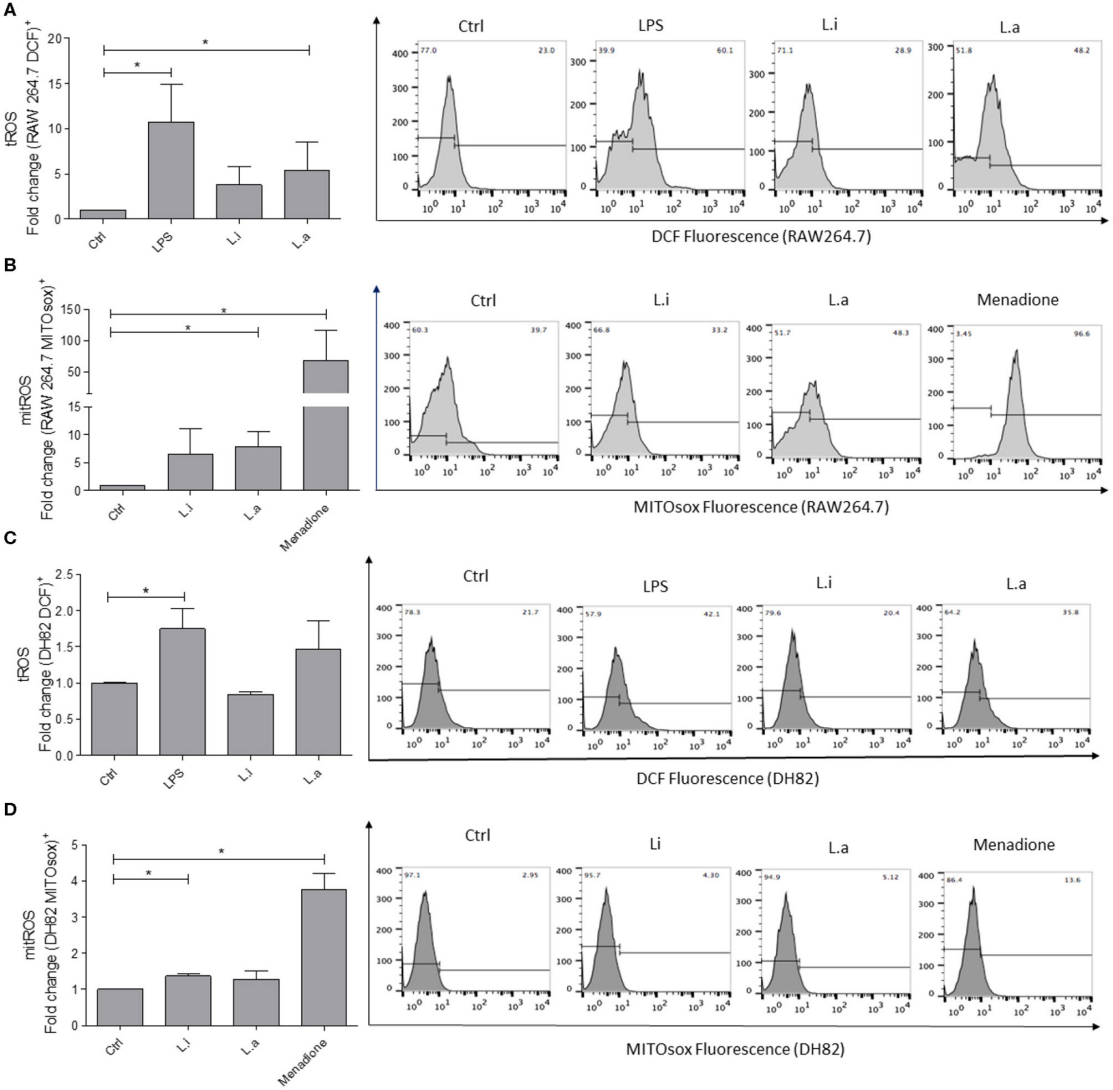
Intracellular ROS production in RAW264.7 murine and DH82 canine macrophages. RAW264.7 **(A)** or DH82 **(C)** macrophages were stimulated or not with 1 μg/mL of LPS or infected with *L. amazonensis* (L.a) or *L. infantum* (L.i) in a ratio of 5p:1m, and total ROS (tROS) production was detected after 24 h with 0.5 mM DCFH-DA. RAW264.7 **(B)** or DH82 **(D)** macrophages stimulated or not with 0.05 mM menadione or infected with *L. amazonensis* (L.a) or *L. infantum* (L.i) as above and mitROS production detected after 24 h with 1 mM MITOsox. Data were normalized as fold change in comparison to control cells (unstimulated cells incubated with DCF or MITOsox) and are presented as mean ± SEM of three independent experiments. *p < 0.05. Representative histograms of %DCF-FM^+^ and MITOSox^+^ cells of unstimulated (Ctrl), stimulated with LPS, or infected with *L. infantum* or *L. amazonensis* are shown. Representative histograms of % of fluorescent cells in unstimulated cells (Ctrl) or LPS- or menadione-stimulated; infected with *L. infantum* or *L. amazonensis* are shown.

Total ROS (tROS) analysis showed that RAW264.7 murine macrophages stimulated with LPS augmented tROS production by 10-fold over the control, and after *L. amazonensis* infection, 5 times more tROS production was detected compared with the control; however, a significant increase in tROS production in *L. infantum*–infected cells was not observed (Figure 5A). Mitochondrial ROS (mitROS) evaluation showed that RAW264.7 murine macrophages, when stimulated with menadione, induced 42-fold more mitROS compared with the control. In *L. amazonensis*–infected cells, an increase of 8 times in mitROS in relation to unstimulated control was observed. However, although mitROS was increased in *L. infantum*–infected RAW264.7 cells, it was not statistically significant (Figure 5B).

Total ROS production by DH82 canine macrophages showed a minor production compared to RAW264.7 murine macrophages. DH82 canine macrophages stimulated with LPS increased 1.7 times tROS production, and in *Leishmania*-stimulated cells, a significant augment in tROS production was not observed (Figure 5C). Mitochondrial ROS analysis showed that DH82 canine macrophages stimulated with menadione used as positive control increased 4-fold mitROS production, while *L. infantum* infection augmented 0.73-fold over mitROS in unstimulated control. Even though *L. amazonensis* infection in RAW264.7 macrophages induced an increase in mitROS, a significant induction of mitROS in *L. amazonensis*–infected DH82 cells was not observed (Figure 5D).

**Figure 6:**
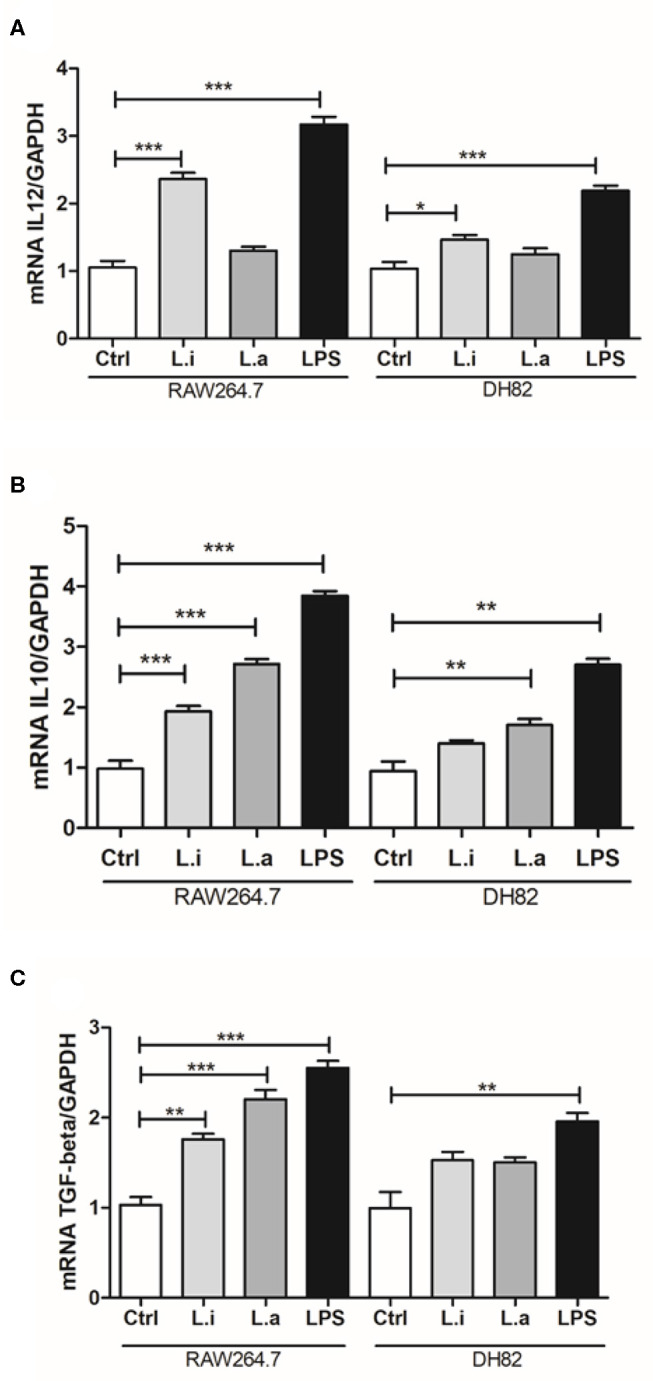
Cytokine profile in *Leishmania*-infected canine (DH82) and murine (RAW264.7) macrophages. Macrophages were stimulated or not (Ctrl) with 1 μg/mL of LPS or infected with *L. amazonensis* or *L. infantum*. After 24 h of infection, the cells were processed and mRNA for the cytokines quantified by qRT-PCR. Data from the experiments were normalized using GADPH primers as an endogenous control. LPS was used as positive control. Data from triplicate are expressed as means ± SEM. *p < 0.05; **p < 0.01; ***p < 0.001.

Next, we determined the cytokine profiles of both macrophages. Our results evidenced that during infection in DH82 cells, *L. infantum* increased 1.4 times IL-12 mRNA expression (Figure 6A) and *L. amazonensis* induced 1.8 times more IL-10 mRNA in relation to uninfected control cells (Figure 6B). In RAW264.7 macrophages, it was observed that *L. infantum* induced 2.2 times more IL-12 mRNA expression (Figure 6A) and both species augmented around 2-fold IL-10 (Figure 6B) and TGF-β mRNA expression compared to control uninfected cells (Figure 6C). In both macrophage cell lines, LPS stimulation increased the mRNA expression of the three cytokines IL-12, IL-10, and TGF-β, but LPS stimulation induced around 1.5 times more IL-12, TGF-β, and IL-10 mRNA expression in RAW 264.7 macrophages than in DH82 cells (Figures 6A–C). IL-6, an important acute phase cytokine, was analyzed only in DH82 cells, and an increase in IL-6 mRNA expression during *L. infantum* infection was observed, but not in *L. amazonensis infection* (Figure S3).

**Figure 7:**
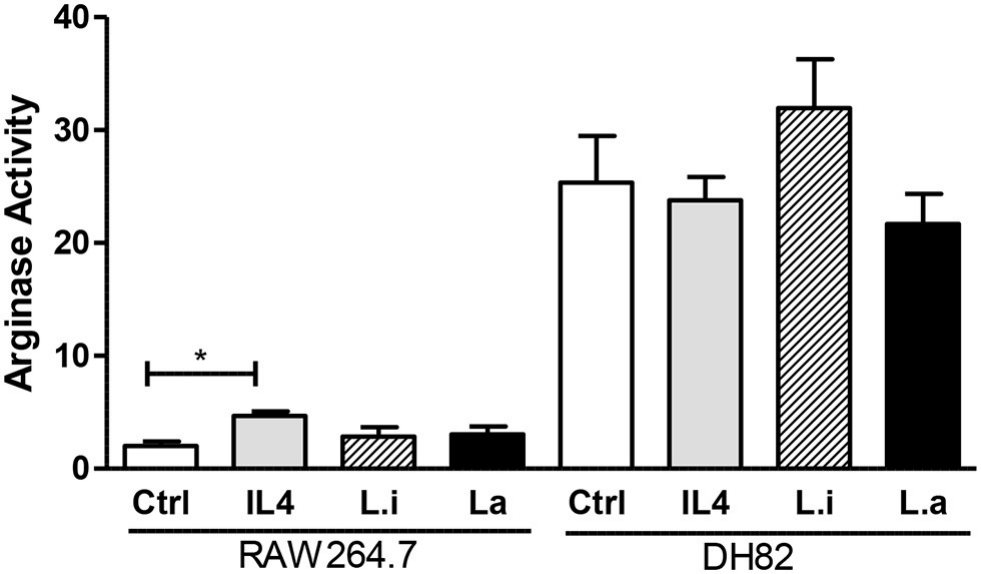
Arginase activity in canine DH82 and murine RAW264.7 macrophages. The cells were infected or not (Ctrl) with *L. infantum* (Li) or *L. amazonensis* (La) (5p:1m) or incubated with 40 ng/mL murine IL-4. After 24 h of infection, the cells were lysed and arginase activity measured by urea concentration at 540 nm. Data from triplicate are expressed as mean ± SEM. *p < 0.05.

Arginase activity analysis showed that uninfected DH82 cells produced 12.5-fold higher arginase activity compared to RAW 264.7 cells and that *Leishmania* infection or IL-4 incubation did not modulate arginase activity in DH82 cells. However, in RAW264.7 cells, IL-4 incubation increased 2.2 times the arginase activity compared to control cells, but similar to observed in *Leishmania* infected DH82 both *Leishmania* species did not modulate arginase activity (Figure 7).

**Figure 8:**
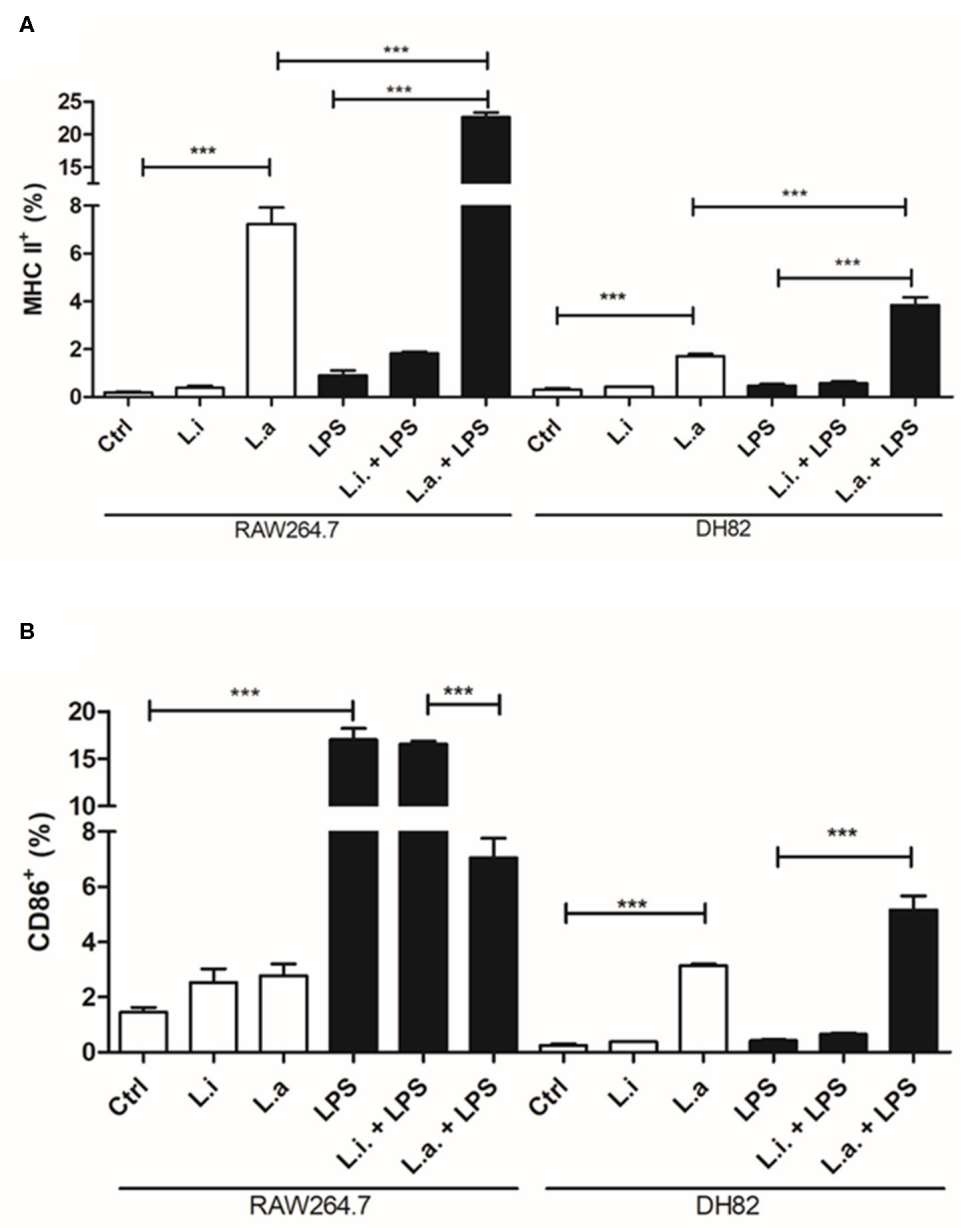
MHC and CD86 expression in canine DH82 and murine RAW264.7 macrophages. The cells (5 × 10^5^/mL) infected or not with (Li.) *L. infantum* or (La.) *L. amazonensis* (5p:1m) were activated or not (Ctrl) with LPS [500 ng/mL] and maintained at 34 or 37°C/5% CO_2_. After 24 h post-infection, the cells were detached, stained for MHC **(A)** and CD86 **(B)** and analyzed by flow cytometry. Data from duplicate are expressed as mean ± SEM. ***p < 0.0001.

The evaluation of MHC and CD86 molecules in macrophages during *Leishmania* infection showed that *L. amazonensis* increased 5.6 and 40 times the MHC expression in DH82 and RAW264.7 cells, respectively; however, *L. infantum* did not modulate MHC expression in any of the macrophages (Figure 8A). Similarly, LPS stimulation alone or together with *L. infantum* did not increase MHC expression in none of the cells, but it potentiated the MHC expression induced by *L. amazonensis*, which increased 2.2 and 3.1 times in DH82 and RAW264.7 macrophages, respectively (Figure 8A). In contrast with *L. infantum*, which did not modulate CD86 expression in DH82 cells, *L. amazonensis* infection increased 15-fold its expression in these macrophages (Figure 8B). Interestingly, in RAW264.7 cells although the expression of CD86 doubled in relation to control after both parasites' stimuli, the increase was not significant. Moreover, CD86 expression was not modulated by LPS stimulation of DH82 cells; nevertheless, it augmented 11.7 times CD86 expression in RAW264.7 macrophages. Interestingly, in the presence of *L. amazonensis*, a reduction of 2.4 times in CD86 expression in LPS-stimulated cells was observed (Figure 8B).

## Discussion

The use of a cell line for studying *in vitro* infections is a useful tool to analyze distinct aspects of *Leishmania* biology. Macrophage cell lines from different origins are favorable to *Leishmania* infection. Thus, the achievement of a canine macrophage–like cell line DH82 prompted us to characterize aspects of the infection with two different species of *Leishmania*. Interestingly, a study comparing the infectivity of different macrophages (mouse, human and dog) by *L. infantum* showed that DH82 canine macrophages, and not the murine macrophages (bone marrow and peritoneal), had a more similar infection when compared to human peripheral blood macrophages (Maia et al., 2007). These reinforce the need to better understand the dynamics and conditions of *Leishmania* infection in a canine cell line, which could be used as experimental model to test drugs, to investigate signaling pathways or modulation of the immune response. Thus, we compared the ability of two different *Leishmania* species to interact with DH82 and RAW264.7 macrophages. It was observed that *L. amazonensis* promastigotes better interacted and were phagocytosed by both canine and murine macrophages, compared to *L. infantum* ones. The ability of DH82 canine macrophages to be infected by a protozoa have been demonstrated using *Trypanosoma cruzi* and *L. infantum* (Maia et al., 2007; Mendonça et al., 2017). Another study comparing infection of Y*ersinia* pestis bacteria between DH82 and RAW264.7 macrophages showed an infection rate of >90% in both macrophages after 2.5 h p.i. (Ponnusamy and Clinkenbeard, 2012). Our data demonstrated that DH82 macrophages were able to interact with *Leishmania* parasites; however, the uptake rate is lower when compared to RAW264.7 murine macrophages, probably by the difference of molecules involved in parasite recognition presented by these cells. Little is known about the molecules expressed by DH82 canine macrophages, although the presence of Fc receptors, CD44 and TLR4, was already detected in these cells (Wellman et al., 1988; Alldinger et al., 1999; Fujimoto et al., 2012); other receptors need to be characterized.

Although the analysis of parasites per cell in 24 and 48 h post-infection showed that the number of parasites in *L. amazonensis*–infected DH82 cells was higher compared with that in RAW264.7 ones, there is no significant difference in the number of parasites per cell in 24 and 48 h in *L. amazonensis*–infected DH82 cells, which suggests that the parasite is not multiplying. The same is observed in RAW 264.7. On the other hand, when we observed the % of infected macrophages, only the point of 48 h was higher in *L. amazonensis*–infected DH82. This may be due to the difference in duplication time of these cells (Barnes et al., 2000; Sakagami et al., 2009; Maeda et al., 2016). Curiously, this difference in % of infected macrophages was not observed in *L. infantum* DH82 interaction compared to RAW264.7 cells. Comparing the data of survival and the parasite load, the different duplication times between these macrophages (Barnes et al., 2000; Sakagami et al., 2009; Maeda et al., 2016) may have influenced the parasite count in stained assays.

The comparison of *L. infantum* survival after 24 h of infection in different macrophages showed that DH82 macrophages and human peripheral blood monocytes infected with *L. infantum* isolated from human and dog samples were less permissive and have a lower number of amastigotes per macrophages compared to mouse bone marrow–derived and mouse peritoneal macrophages (Maia et al., 2007). In our data, the parasite survival analysis of *L. amazonensis* showed a reduction on parasite after 48 h of interaction in both macrophages; however, this reduction was minor in DH82 macrophages compared to RAW264.7 ones. The same was observed in *Leishmania infantum* survival inside both macrophages. No difference in the number of parasites per cell was observed in both macrophages in 24 or 48 h, indicating that the parasite is not multiplying. A study using canine peritoneal macrophages showed that *L. amazonensis, L. braziliensis, and L. infantum* infect the cells and after 24 h of infection the difference on the % of infected cells disappeared (Madeira et al., 1999). Although *L. infantum* is implicated in canine leishmaniasis, the detection of other species of *Leishmania* in naturally infected dogs has been reported (Sanches et al., 2016; Valdivia et al., 2017; Alves Souza et al., 2019).

It has long been described that macrophages produce reactive species, such as superoxide (${\text{O}}_{2}^{-}$) anion, hydrogen peroxide (H_2_O_2_), and also nitric oxide (NO) in high quantities as an efficient mechanism to control intracellular pathogens (Gantt et al., 2001; Piacenza et al., 2019). NO production analysis showed that DH82 macrophages failed to increase the number of DAF^+^ macrophages after any stimuli used, such as LPS, *L. amazonensis*, or *L. infantum*. Similarly, the production of NO was also evaluated in DH82 macrophages during *Trypanosoma cruzi* infection, and an increase in this mediator was also not observed after infection or stimulus with LPS and IFN-γ (Mendonça et al., 2017). Curiously, Wasserman et al. (2012) showed that unstimulated DH82 canine cells presented around 90 μM of nitrite detected by the Griess reaction, but the cells failed to respond when it was stimulated with LPS from 0.1 to 10 μg/mL. On the other hand, RAW264.7 murine macrophages augmented NO production, when stimulated by LPS and during *L. amazonensis* and *L. infantum* infection, reaching up to 60% of the cell population expressing NO during *L. amazonensis* infection.

The same profile was observed in ROS production by both cells. *L. amazonensis* infection induced tROS production by RAW264.7, but not by DH82 cells. On the other hand, *L. infantum* infection did not induce ROS production by none of the two macrophages tested, and yet the tROS as well as mitROS quantity observed in DH82 cells was lower than in RAW264.7 ones. Recently, the involvement of ROS production in inducing inflammasome activation and restricting *L. amazonensis* infection was demonstrated in murine bone marrow–derived macrophages (Lima-Junior et al., 2013, 2017). A study about IL-1 maturation showed that RAW264.7 murine macrophages did not have apoptotic speck-like protein with a caspase-activating recruiting domain for an efficient inflammasome activation (Pelegrin et al., 2008). It is not known if DH82 canine macrophages have all the proteins to assemble an efficient inflammasome, and further studies should be done to clarify this issue.

The balance of arginase and NO synthase in the control of *Leishmania* growth is well-established (Green et al., 1990; Gantt et al., 2001; Iniesta et al., 2001; Van Assche et al., 2011). Interestingly, when we analyzed arginase activity in both macrophages, it was observed that uninfected or unstimulated DH82 canine macrophages have higher arginase activity compared to RAW264.7 cells and their stimulation with IL-4 or *Leishmania* infection did not increase it. On the contrary, RAW264.7 murine macrophages have lower arginase activity which was increased after IL-4 incubation; however, *Leishmania* did not modulate it. The importance of arginase activity in the proliferation of *Leishmania* has been demonstrated (Iniesta et al., 2001), since its inhibition augments *Leishmania* killing by macrophages.

The difference in NO and ROS production and arginase activity between DH82 and RAW264.7 macrophages may explain the infection rates observed. Although the initial association rate (2 h) of *L. amazonensis* with both macrophages was similar, the infection rate at 48 h in RAW264.7 cells was significantly lower than in DH82 ones. Nevertheless, when we evaluated the parasite load after 48 h of infection, it was similar in both macrophages suggesting that other mechanisms should be involved in this latter phenomenon, which still needs to be investigated.

Cytokines are key mediators for determining the modulation of the immune response; thus, it is essential to characterize cytokines produced by macrophages during *Leishmania* infection. Few works demonstrated cytokine profile in *Leishmania*-infected RAW264.7 murine macrophages. The production of IL-10 in the supernatant of *L. amazonensis*–infected RAW264.7 cells was observed after 72 h of infection and the use of an antibody to abrogate it, preventing *L. amazonensis* proliferation (Pereira et al., 2010). In *L. infantum*–infected RAW 264.7 cells, the presence of IL-10 in culture supernatants at 24 h post-infection was also reported (Figueiredo et al., 2017). Our results corroborated that, since we observed IL-10 RNAm expression in RAW264.7 murine macrophages infected by both species. Besides, *L. infantum* infection also induced TGF-β and IL-12 mRNA expression. In the murine model of infection, an inhibition of IL-6 production in *L. amazonensis*–infected macrophages was observed (Craig et al., 2017). Our data showed that *L. infantum* induced IL-6 mRNA expression in canine DH82 macrophages, although it is a cytokine of acute phase produced by different cell types; this cytokine is involved in Th2 differentiation, which could influence the arginase activity (Diehl and Rincón, 2002).

For the first time, our work evaluated the expression of cytokines in *Leishmania*-infected DH82 canine macrophages, and similarly to *L. amazonensis*–infected RAW264.7 cells we also observed an increase in the expression of IL-10 mRNA in canine DH82 macrophages; however, *L. infantum*–infected DH82 cells induced IL-12 mRNA. None of the *Leishmania* species we tested induced TGF-β in DH82 canine macrophages in a significant manner, although an upward trend was observed. The cytokine profile in *T. cruzi*–infected DH82 cells showed the presence of IL-10, TGF-β, and TNF-α (Mendonça et al., 2017). The induction of IL-10 during *L. amazonensis* infection could explain the high amount of parasite observed at 48 h post-infection in comparison to *L. infantum*.

MHC and co-stimulatory molecules are important signals required by T cells to be activated, and down-modulation of these molecules is an escape mechanism of the immune response. Actually, it was already shown that *L. amazonensis* and *L. mexicana* amastigotes inside the parasitophorous vacuole degrade MHC class II molecules (De Souza Leao et al., 1995; Antoine et al., 1999; Costa et al., 2018), without affecting CD86 expression (Costa et al., 2018). Nevertheless, a study in canine peripheral blood monocyte–derived macrophages showed an upregulation of MHC molecules in cells infected with *L. infantum* in the presence of T cells *in vitro* (Diaz et al., 2012). Here we did not observe induction of MHC or CD86 expression in none of the cell lines during *L. infantum* infection. Meanwhile, *L. amazonensis* induced MHC expression in both cell lines. Interestingly, *L. amazonensis* downregulated CD86 expression induced by LPS in RAW264.7 cells. Interestingly, ROS and NO production is also not stimulated in DH82 by LPS, showing a low responsiveness to this stimulant by these cells in relation to RAW264.7 cells. Synergism and higher MHC expression were obtained after stimulation of both cell lines by LPS and *L. amazonensis* compared to each of the isolated stimuli. Remarkably, increased MHC and CD80 expression in DH82 cells was obtained upon stimulation with LPS plus IFN-γ (Mendonça et al., 2017). Recently, it was shown that the DH82 cell line is able to differentiate in the M2a subtype upon IL-4 and IL-13 human cytokine stimulation. The cells did not augment CD206 expression, but express FCεRI independent of stimuli, and upregulated CD163 and IL-10 gene expression. Our results showed that these cells express low levels of CD86 and MHC after LPS and/or *Leishmania* infection compared to RAW264.7 murine cells. Besides having high arginase activity, all this configures that this cell line could be an M2 subtype macrophage model to study *Leishmania* infection.

Taken together, our results show that although DH82 canine and RAW264.7 murine macrophages present different infection profiles after the initial *Leishmania* infection and differently modulate the macrophage response, after 48 h of infection, the parasite load profile is similar. Further studies are necessary in order to elucidate other possible mechanisms involved in *Leishmania* immunomodulation of these macrophage lines.

## Data Availability Statement

All datasets generated for this study are included in the article/Supplementary Material.

## Author Contributions

NN, LS, AW, DD-R, CF, UG, ES, and LP conceived and designed the experiments. NN, LS, RS, IL, DS, ÁV, AW, and LP performed the experiments. NN, RS, LS, IL, ÁV, DD-R, ES, CF, and LP analyzed the data. LP and NN wrote the paper.

## Conflict of Interest

The authors declare that the research was conducted in the absence of any commercial or financial relationships that could be construed as a potential conflict of interest.

## Supplementary Material

Figure S1 Representative dotplots of macrophages. Dot plots of DH82 **(A)** and RAW264.7 **(B)** macrophages showing the gates used to acquire and analyze the macrophages populations. Data representative of three independent experiments.

Figure S2 Optical microscopy DH82 canine macrophages infected with *L. amazonensis* **(A,C)** and *L. infantum* **(B,D)** for 24 and 48 h post-infection Giemsa stained.

Figure S3 IL-6 detection in *Leishmania* infected-DH82 macrophages. Macrophages were stimulated or not (Ctrl) with 1 μg/mL of LPS or infected with *L. amazonensis* or *L. infantum* in a ratio of 5p:1m. After 24 h of infection, the cells were processed and mRNA quantification by qRT-PCR. Data were normalized using GADPH primers as an endogenous control, and LPS was used as positive control. Data are expressed as means ± SEM of triplicate. ^***^*p* < 0.001.

## References

[B1] Alldinger S. Baumgärtner W. Kremmer E. Fonfara S. (1999). Characterization of a canine CD44 specific monoclonal antibody. Transbound. Emerg. Dis. 46, 19–32. 10.1046/j.1439-0442.1999.00184.x 10085764

[B2] Alves Souza N. Souza Leite R. de Oliveira Silva S. Groenner Penna M. Figueiredo Felicori Vilela L. Melo M. N.. (2019). Detection of mixed Leishmania infections in dogs from an endemic area in southeastern Brazil. Acta Tropica.193:12–17. 10.1016/j.actatropica.2019.02.016 30772331

[B3] Antoine J. C. Lang T. Prina E. Courret N Hellio R. (1999). H-2M molecules, like MHC class II molecules, are targeted to parasitophorous vacuoles of Leishmania-infected macrophages and internalized by amastigotes of *L. amazonensis* and *L. mexicana*. J Cell Sci. 112, 2559–2570. 10393812 10.1242/jcs.112.15.2559

[B4] Barnes A. Bee A. Bell S. Gilmore W. Mee A. Morris R.. (2000). Immunological and inflammatory characterisation of three canine cell lines: K1, K6 and DH82. Vet. Immunol. Immunopathol. 30, 9–25. 10.1016/S0165-24270000184-7 10889296

[B5] Castro E. A. Thomaz-Soccol V. Augur C. Luz E. (2007). Leishmania (Viannia) Braziliensis: epidemiology of canine cutaneous Leishmaniasis in the State of Paraná (Brazil). Exp. Parasitol. 11, 13–21. 10.1016/j.exppara.2007.03.003 17449032

[B6] Channon J. Y. Roberts M. B. Blackwell J. M. (1984). A study of the differential respiratory burst activity elicited by promastigotes and amastigotes of *Leishmania donovani* in murine resident peritoneal macrophages. Immunol. 53, 345–355. 6490087 PMC1454813

[B7] Costa S. S. Fornazim M. C. Nowill A. E. Giorgio S. (2018). Leishmania amazonensis induces modulation of costimulatory and surface marker. Parasit. Immunol. 40:e12519. 10.1111/pim.12519 29380393

[B8] Craig E. Huyghues-Despointes C. E. Yu C. Handy E. L. Sello J. K. Kima P. E. (2017). Structurally optimized analogs of the retrograde trafficking inhibitor Retro-2cycl limit Leishmania infections. PLoS. Negl. Trop. Dis. 11:e0005556. 10.1371/journal.pntd.0005556 28505157 PMC5444862

[B9] Dantas-Torres F. Solano-Gallego L. Baneth G. Ribeiro V.M. dePaiva-Cavalcanti M. Otranto D. (2012). Canine leishmaniasis in the old and new worlds: unveiled similarities and differences. Trends Parasitol. 28, 531–538. 10.1016/j.pt.2012.08.007 22995719

[B10] De Souza Leao S. Lang T. Prina E. Hellio R. Antoine J. C. (1995). Intracellular Leishmania amazonensis amastigotes internalize and degrade MHC class II molecules of their host cells. J. Cell Sci. 108, 3219–3231. 7593283 10.1242/jcs.108.10.3219

[B11] Diaz S. da Fonseca I. P. Rodrigues A. Martins C. Cartaxeiro C. Silva M. J.. (2012). Canine leishmaniosis. Modulation of macrophage/lymphocyte interactions by *L. infantum*. Vet. Parasitol. 189, 137–144. 10.1016/j.vetpar.2012.05.004 22698797

[B12] Diehl S. Rincón M. (2002). The two faces of IL-6 on Th1/Th2 differentiation. Mol Immunol. 39, 531–536. 10.1016/S0161-5890(02)00210-9 12431386

[B13] Figueiredo W. M. E. Viana S. M. Alves D. T. Guerra P. V. Coêlho Z. C. B. Barbosa H. S.. (2017). Protection mediated by chemokine CXCL10 in BALB/c mice infected by *Leishmania infantum*. Mem. Inst. Oswaldo Cruz. 112, 561–568. 10.1590/0074-02760160529 28767981 PMC5530548

[B14] Fujimoto Y. Nakatani N. Kubo T. Semi Y. Yoshida N. Nakajima H.. (2012). Adenosine and ATP affect LPS-induced cytokine production in canine macrophage cell line DH82 cells. J. Vet. Med. Sci. 74, 27–34. 10.1292/jvms.11-0228 21873810

[B15] Gantt K. R. Goldman T. L. McCormick M. L. Miller M. A. Jeronimo S. M. Nascimento E. T.. (2001). Oxidative responses of human and murine macrophages during phagocytosis of *Leishmania chagasi*. J. Immunol. 167, 893–901. 10.4049/jimmunol.167.2.893 11441096

[B16] Gomes N. A. Gattass C. R. Barreto-De-Souza V. Wilson M. E. DosReis G. A. (2000). TGF-beta mediates CTLA-4 suppression of cellular immunity in murine Kalaazar. J. Immunol. 164, 2001–2008. 10.4049/jimmunol.164.4.2001 10657651

[B17] Gontijo B. De Carvalho M. L. R. (2003). Leishmaniose Tegumentar Americana. Rev. Soc. Bras. Med. Trop. 36, 71–80. 10.1590/S0037-86822003000100011 12715066

[B18] Green S. J. Meltzer M. S. Hibbs J. B. Jr. Nacy C. A. (1990). Activated macrophages destroy intracellularLeishmania major amastigotes by an L-arginine-dependent killing mechanism. J. Immunol. 144, 278–283. 2104889

[B19] Handman E. Bullen D. V. (2002). Interaction of Leishmania with the host macrophage. Trends Parasit. 8, 332–334. 10.1016/S1471-4922(02)02352-8 12377273

[B20] Horta M. F. Mendes B. P. Roma E. H. Noronha F. S. M. Macedo J. P. Oliveira L. S.. (2012). Reactive oxygen species and nitric oxide in cutaneous Leishmaniasis. J. Parasitol. Res. 2012:203818. 10.1155/2012/203818 22570765 PMC3337613

[B21] Howerth E. W. Parlavantzas G. S. Stallknecht D. E. (2004). Replication of epizootic haemorrhagic disease and bluetongue viruses in DH82 cells. Vet. Ital. 40, 520–524. 20422580

[B22] Iniesta V. Gómez-Nieto L. C. Corraliza I. (2001). The inhibition of arginase by N(omega)-hydroxy-l-arginine controls the growth of Leishmania inside macrophages. J. Exp. Med. 193, 777–784. 10.1084/jem.193.6.777 11257143 PMC2193414

[B23] Lima-Junior D. S. Costa D. L. Carregaro V. Cunha L. D. Silva A. L. Mineo T. W.. (2013). Inflammasome-derived IL-1β production induces nitric oxide-mediated resistance to Leishmania. Nat. Med. 19, 909–915. 10.1038/nm.3221 23749230

[B24] Lima-Junior D. S. Mineo T. W. P. Calich V. L. G. Zamboni D. S. (2017). Dectin-1 Activation during *Leishmania amazonensis* phagocytosis prompts Syk-dependent reactive oxygen species production to trigger inflammasome assembly and restriction of parasite replication. J. Immunol. 199, 2055–2068. 10.4049/jimmunol.1700258 28784846

[B25] Madeira M. Barbosa-Santos E. Marzochi M. (1999). Experimental infection of canine peritoneal macrophages with visceral and dermotropic Leishmania strains. Mem. Inst. Oswaldo. Cruz. 94, 645–648. 10.1590/s0074-02761999000500015 10464409

[B26] Maeda J. Froning C. E. Brents C. A. Rose B. J. Thamm D. H. Kato T. A. (2016). Intrinsic radiosensitivity and cellular characterization of 27 canine cancer cell lines. PLoS ONE. 11:e0156689. 10.1371/journal.pone.0156689 27257868 PMC4892608

[B27] Maia C. Rolão N. Nunes M. Gonçalves L. Campino L. (2007). Infectivity of five different types of macrophages by *Leishmania infantum*. Acta Tropica 103, 150–155. 10.1016/j.actatropica.2007.06.001 17626776

[B28] Mendonça P. H. B. da Rocha R. F. D. B. Moraes J. B. de B LaRocque-de-Freitas I. F. Logullo J. Morrot A.. (2017). Canine macrophage DH82 cell line as a model to study susceptibility to *Trypanosoma cruzi* infection. Front. Immunol. 8:604. 10.3389/fimmu.2017.00604 28620374 PMC5449653

[B29] Moreira N. das D. Vitoriano-Souza J. Roatt B.M. Vieira P.M. de A Ker H.G Cardoso J.M. de O. Giunchetti R.C.. (2012). Parasite burden in hamsters infected with two different strains of Leishmania (Leishmania) infantum: “Leishman Donovan units” versus real-time PCR. PloS One 7:e47907. 10.1371/journal.pone.0047907 23112869 PMC3480442

[B30] Moreira N. das D. Vitoriano-Souza J. Roatt B. M. Vieira P. M. de A. Coura-Vital W. Cardoso J. M. de O.. (2016). Clinical, hematological and biochemical alterations in hamster (*Mesocricetus auratus*) experimentally infected with *Leishmania infantum* through different routes of inoculation. Parasit. Vectors 9:181. 10.1186/s13071-016-1464-y 27030128 PMC4815141

[B31] Mosser D. M. Rosenthal L. A. (1993). Leishmania-macrophage interactions: multiple receptors, multiple ligands and diverse cellular responses. Semin. Cell Biol. 4, 315–322. 10.1006/scel.1993.1038 8257783

[B32] Naderer T. McConville M. J. (2008). The Leishmania-macrophage interaction: a metabolic perspective. Cell. Microbiol. 10, 301–308. 10.1111/j.1462-5822.2007.01096.x 18070117

[B33] Paz G. F. Rugani J. M. N. Marcelino A. P. Gontijo C. M. F. (2018). Implications of the use of serological and molecular methods to detect infection by *Leishmania* spp. in urban pet dogs. Acta Tropica 182, 198–201. 10.1016/j.actatropica.2018.03.018 29545151

[B34] Pelegrin P. Barroso-Gutierrez C. Surprenant A. (2008). P2X7 receptor differentially couples to distinct release pathways for IL-1beta in mouse macrophage. J. Immunol. 180, 7147–7157. 10.4049/jimmunol.180.11.7147 18490713

[B35] Pereira R. M. Teixeira K. L. Barreto de Souza V. Calegari-Silva T. C. De-Melo L. D. Soares D. C.. (2010). Novel role for the double-stranded RNA-activated protein kinase PKR: modulation of macrophage infection by the protozoan parasite Leishmania. FASEB J. 24, 617–626. 10.1096/fj.09-140053 19812373

[B36] Piacenza L. Trujillo M. Radi R. (2019). Reactive species and pathogen antioxidant networks during phagocytosis. J. Exp. Med. 216, 501–516. 10.1084/jem.20181886 30792185 PMC6400530

[B37] Ponnusamy D. Clinkenbeard K. D. (2012). Yersinia pestis intracellular parasitism of macrophages from hosts exhibiting high and low severity of plague. PLoS ONE 7:e42211. 10.1371/journal.pone.0042211 22848745 PMC3407133

[B38] Ribeiro-Gomes F. L. Moniz-de-Souza M. C. A Alexandre-Moreira M. S. Dias W. B. Lopes M. F. Nunes M. P.. (2007). Neutrophils activate macrophages for intracellular killing of Leishmania major through recruitment of TLR4 by neutrophil elastase. J. Immunol. 179, 3988–3994. 10.4049/jimmunol.179.6.3988 17785837

[B39] Ribeiro-Gomes F. L. Silva M. T. Dosreis G. A. (2006). Neutrophils, apoptosis and phagocytic clearance: an innate sequence of cellular responses regulating intramacrophagic parasite infections. Parasitology 132, S61–S68. 10.1017/S0031182006000862 17018166

[B40] Sakagami H. Kishimo K. Amano O. Kanda Y. Kuni S. Yokote Y.. (2009). Cell death induced by nutritional starvation in mouse macrophage-like RAW 264.7 cells. Anticancer Res. 29, 343–348. 19331171

[B41] Sanches L. D. Martini C. C. Nakamura A. A. Santiago M. E. Dolabela de Lima B. Lima V. M. (2016). Natural canine infection by Leishmania infantum and Leishmania amazonensis and their implications for disease control. Rev. Bras. Parasitol. Vet. 25, 465–469. 10.1590/s1984-29612016071 27925065

[B42] Valdivia H. O. Almeida L. V. Roatt B. M. Reis-Cunha J. L. Pereira A. A. S. Gontijo C.. (2017). Comparative genomics of canine-isolated *Leishmania* (Leishmania) *amazonensis* from an endemic focus of visceral Leishmaniasis in Governador Valadares, Southeastern Brazil. Sci. Rep. 7:40804. 10.1038/srep40804 28091623 PMC5238499

[B43] Van Assche T. Deschacht M. da Luz R. A. Maes L. Cos P. (2011). Leishmania-macrophage interactions: insights into the redox biology. Free Radic. Biol. Med. 51, 337–351. 10.1016/j.freeradbiomed.2011.05.011 21620959

[B44] Wasserman J. Diese L. VanGundy Z. London C. Carson W. E. Papenfuss T. L. (2012). Suppression of canine myeloid cells by soluble factors from cultured canine Tumor cells. Vet. Immunol. Immunopathol. 145, 420–430. 10.1016/j.vetimm.2011.12.018 22244518 PMC4278197

[B45] Wellman M. L. Krakowka S. Jacobs R. M. Kociba G. J. (1988). A macrophage-monocyte cell line from a dog with malignant histiocytosis. In Vitro Cell. Dev. Biol. J. Tiss. Cult. Assoc. 24, 223–229. 10.1007/BF02623551 3350786

