# Supplementary figures and images for: DH82 Canine and RAW264.7 Murine Macrophage Cell Lines Display Distinct Activation Profiles Upon Interaction With Leishmania infantum and Leishmania amazonensis
Source: Front Cell Infect Microbiol. 2020 Jun 12;10:247. doi: 10.3389/fcimb.2020.00247 (PMC7303514; doi:10.3389/fcimb.2020.00247)

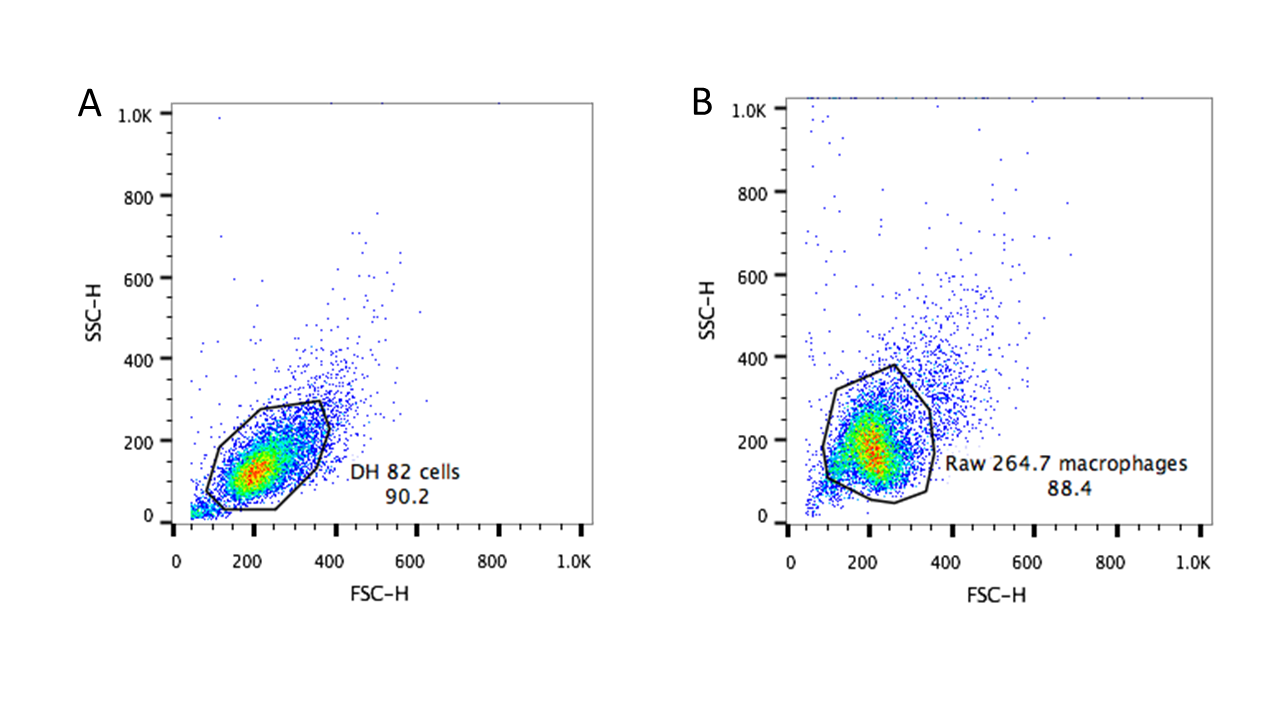

Supplement: Figure S1 — Representative dotplots of macrophages. Dot plots of DH82 (A) and RAW264.7 (B) macrophages showing the gates used to acquire and analyze the macrophages populations. Data representative of three independent experiments. [file Image_1.TIF]

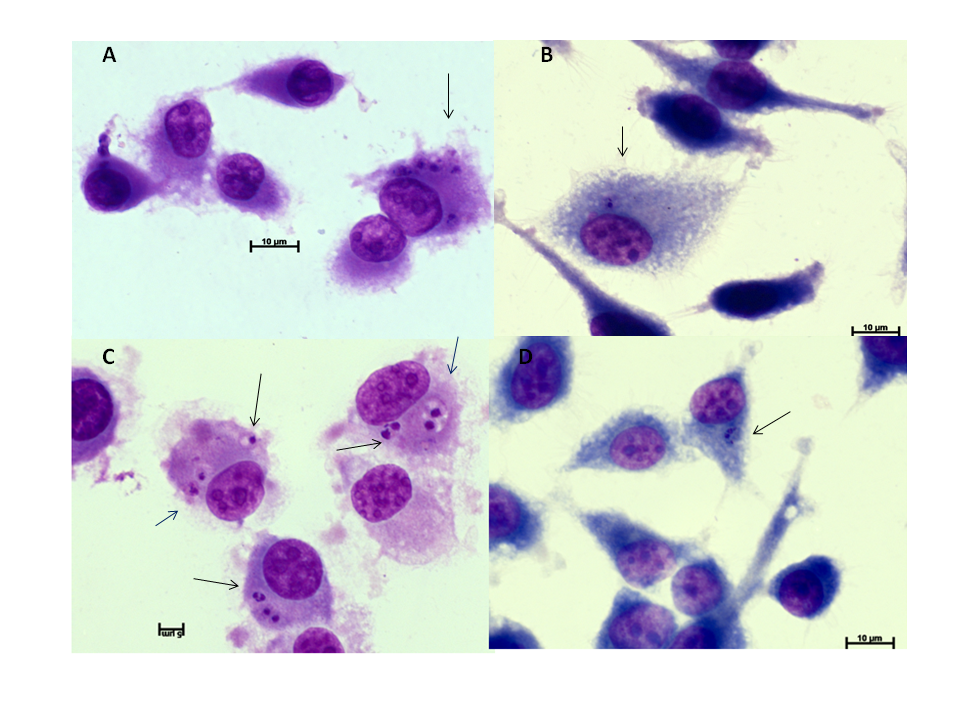

Supplement: Figure S2 — Optical microscopy DH82 canine macrophages infected with L. amazonensis (A,C) and L. infantum (B,D) for 24 and 48 h post-infection Giemsa stained. [file Image_2.TIF]

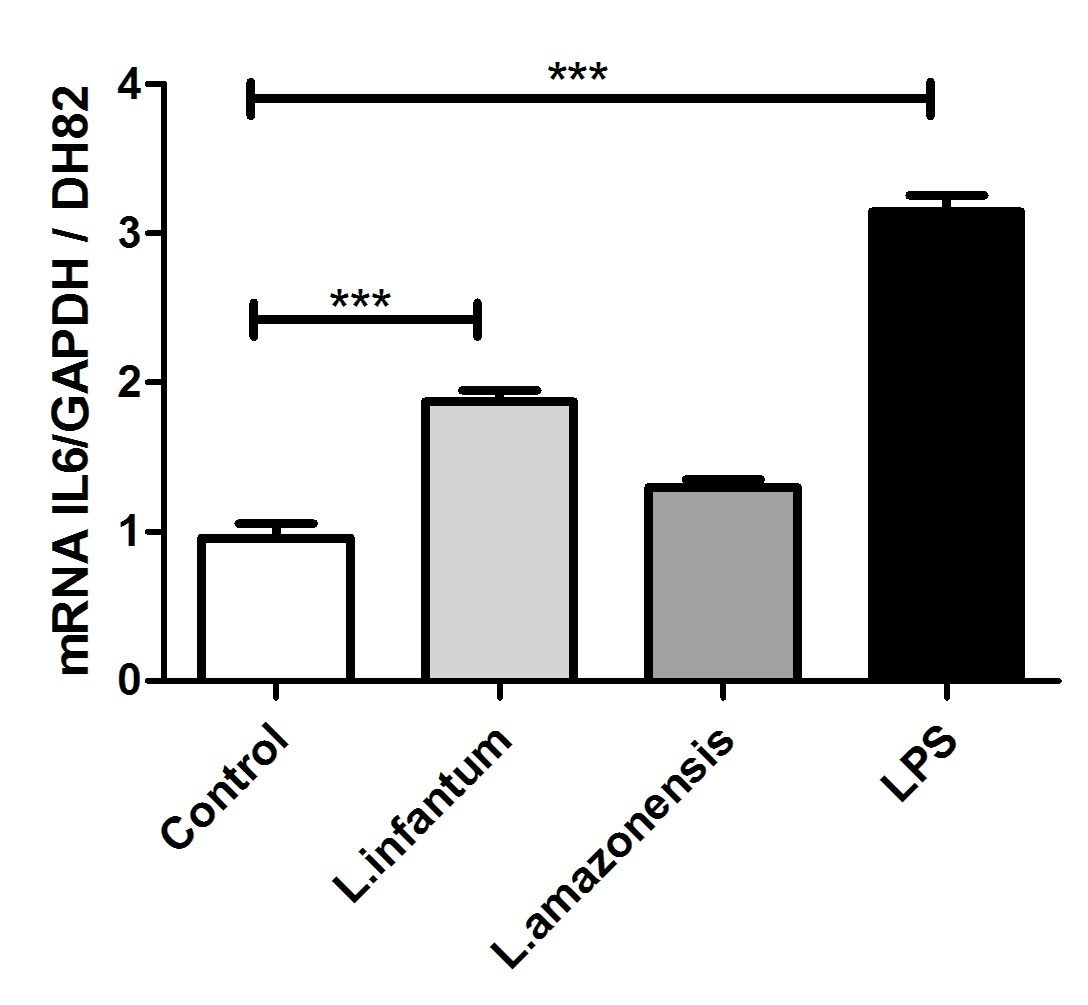

Supplement: Figure S3 — IL-6 detection in Leishmania infected-DH82 macrophages. Macrophages were stimulated or not (Ctrl) with 1 μg/mL of LPS or infected with L. amazonensis or L. infantum in a ratio of 5p:1m. After 24 h of infection, the cells were processed and mRNA quantification by qRT-PCR. Data were normalized using GADPH primers as an endogenous control, and LPS was used as positive control. Data are expressed as means ± SEM of triplicate. ***p < 0.001. [file Image_3.TIF]
